# Supplementary material for: The effect of intrapartum prolonged oxygen exposure on fetal metabolic status: secondary analysis from a randomized controlled trial
Source: Front Endocrinol (Lausanne). 2023 Jun 27;14:1204956. doi: 10.3389/fendo.2023.1204956 (PMC10335765; doi:10.3389/fendo.2023.1204956)
Supplement: Supplementary file 3 [file DataSheet_3.zip › Result-X101SC21092977-Z01-J001-B1-42/3.MetDiffScreening/O.vs.A/O.vs.A_pos_PLSDA-valid.pdf]

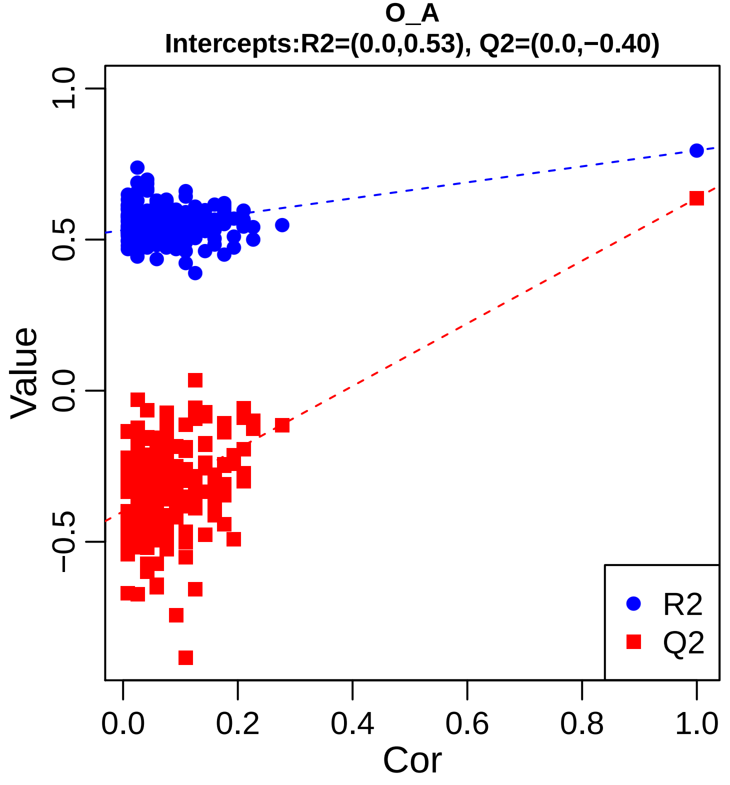
O_A
Intercepts:R2=(0.0,0.53), Q2=(0.0,−0.40)
Value
Cor
1.0
0.5
0.0
−0.5
0.0
0.2
0.4
0.6
0.8
1.0
R2
Q2
